# Activity-Guided Isolation of Bioactive Constituents with Antinociceptive Activity from *Muntingia calabura* L. Leaves Using the Formalin Test

**DOI:** 10.1155/2013/715074

**Published:** 2013-11-21

**Authors:** Mohd. Izwan Mohamad Yusof, Mohd. Zaki Salleh, Teh Lay Kek, Norizan Ahmat, Nik Fatini Nik Azmin, Zainul Amiruddin Zakaria

**Affiliations:** ^1^Integrative Pharmacogenomics Institute, Universiti Teknologi MARA, 42300 Puncak Alam, Selangor, Malaysia; ^2^Faculty of Pharmacy, Universiti Teknologi MARA, 42300 Puncak Alam, Selangor, Malaysia; ^3^Faculty of Applied Sciences, Universiti Teknologi MARA, 40450 Shah Alam, Selangor, Malaysia; ^4^Department of Biomedical Science, Faculty of Medicine and Health Sciences, Universiti Putra Malaysia, 43400 Serdang, Selangor, Malaysia

## Abstract

The present study was conducted to determine the antinociceptive potential of methanol extract of *Muntingia calabura *L. (MEMC) and to isolate and identify the bioactive compound(s) responsible for the observed antinociceptive activity. The MEMC and its partitions (petroleum ether (PEP), ethyl acetate (EAP), and aqueous (AQP) partitions), in the dose range of 100, 500, and 1000 mg/kg, were tested using the formalin-induced nociceptive test. The PEP, which exerted the most effective activity in the respective early and late phase, was further subjected to the fractionation procedures and yielded seven fractions (labelled A to G). These fractions were tested, at the dose of 300 mg/kg, together with distilled water or 10% DMSO (negative controls); morphine and aspirin (positive controls) for potential antinociceptive activity. Of all fractions, Fraction D showed the most significant antinociceptive activity, which is considered as equieffective to morphine or aspirin in the early or late phase, respectively. Further isolation and identification processes on fraction D led to the identification of three known and one new compounds, namely, 5-hydroxy-3,7,8-trimethoxyflavone (**1**), 3,7-dimethoxy-5-hydroyflavone (**2**), 2′,4′-dihydroxy-3′-methoxychalcone (**3**), and **calaburone** (**4**). At the dose of 50 mg/kg, compound **3** exhibited the highest percentage of antinociceptive activity in both phases of the formalin test. In conclusion, the antinociceptive activity of MEMC involved, partly, the synergistic activation of the flavonoid types of compounds.

## 1. Introduction

*Muntingia calabura *L., also known as “*kerukup siam*” and “*buah ceri kampung*” to the Malay, is a flowering plant of the family Elaeocarpaceae [1]. It is native to Central America, western South America, southern Mexico, and the Caribbean but is wildly cultivated in the region of Southeast Asia, including Malaysia. Although this plant did not find its place in the Malay's traditional medicine, it has been claimed by the Peruvian to possess several medicinal values [1, 2]. The leaves of *M. calabura*, in particular, are used to treat pain related to headache, cold, and gastric ulcer, or to attenuate the prostate gland swelling [1–4]. Other parts of *M. calabura*, namely, the roots and flowers, are also claimed to possess medicinal values in Vietnam and Philippines, and used as emmenagogue, abortifacient, antidyspeptic, antispasmodic, and diaphoretic, and to treat headaches, dyspepsia, and spasm [5].

 Various attempts have been made to scientifically demonstrate the medicinal potential of *M. calabura*. The leaves, in particular, have been scientifically demonstrated to exert various pharmacological activities, namely, antitumour, antinociceptive, anti-inflammatory, antipyretic, antibacterial, antiproliferative, and antioxidant activities of *M. calabura *leaves [3, 5–11]. Attempt to determine the phytochemical constituents of the leaves has lead to the identification of several classes of bioactive compounds, namely, flavonoids, tannins, triterpenes, saponins, and steroids, but no alkaloids [8] while further phytochemical study on the MEMC led to the detection of only flavonoids, tannins, and saponins [11]. We have recently reported that the antinociceptive activity of MEMC involves inhibition of the peripheral and central nociceptive pathways, and modulation of the opioid receptors and NO/cGMP pathway [12]. Therefore, in the present study, we attempt to isolate and identify the bioactive compound(s) responsible for the MEMC-induced antinociceptive activity.

## 2. Materials and Methods

### 2.1. Plants Material

The leaves of *M. calabura *were collected from its natural habitat in Shah Alam, Selangor, Malaysia in January 2008. The leaves have been reidentified by a botanist, Dr. Shamsul Khamis, from the Institute of Bioscience (IBS), Universiti Putra Malaysia (UPM), Serdang, Selangor, Malaysia. A voucher specimen (SK 1095/05) has been deposited at the Herbarium of the laboratory of Natural product, Institute of Bioscience, UPM, Selangor, Malaysia.

### 2.2. Preparation of *M. calabura* Extract and Partitions

**Figure 1:**
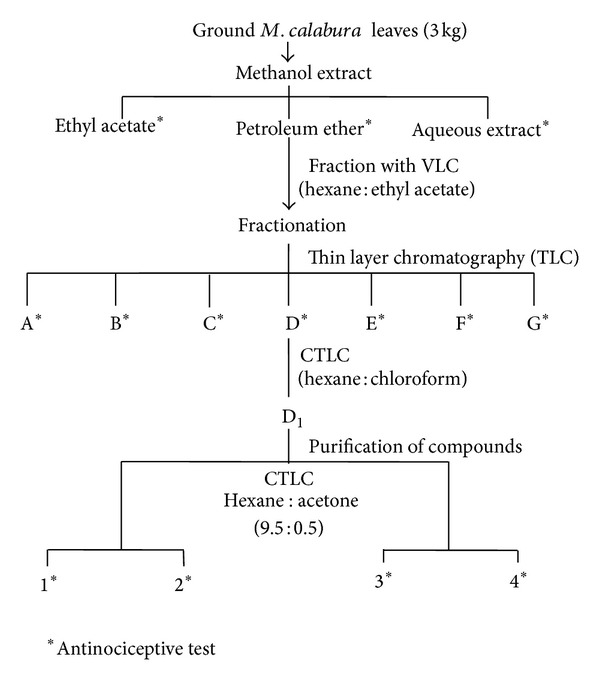
Activity-guided isolation of the antinociceptive-induced chemical constituents of the *Muntingia calabura*.

The extraction procedure is as described by Sani et al. [12] (see Figure 1). *M. calabura* powder (3 Kg) was soaked into 6000 L methanol (MeOH) in the ratio of 1 : 20 (w/v). These mixtures were then left for 72 hours at room temperature and the supernatant was collected and filtered using no. 1 filter paper (Whatman, England). Subsequently, the solvent in the extract was removed under reduced pressure with the rotary evaporator (Rotavapor R-210, Büchi, Switzerland) at 40°C to obtain the crude methanol extract of *M. calabura* (MEMC; X.Y g). The MEMC was then dissolved in 10% DMSO to the requisite doses (100, 500, and 1000 mg/kg) before use and then subjected to the antinociceptive assays.

The preparation of partitions from MEMC was carried out by sequential extraction of the crude dried extract with solvents of different polarity, namely, petroleum ether, ethyl acetate, and distilled water (dH_2_O), in the ratio of 1 : 20 (w/v). The partitioning processes were repeated three times until no more changes in the colour of the supernatant were observed. Each partition obtained, namely, petroleum ether partition (PEP), ethyl acetate partition (EAP), and aqueous partition (AQP), was dissolved in 10% DMSO to the requisite doses (100, 500, and 1000 mg/kg) before being used and then subjected to the antinociceptive assays. The most effective partition, namely, PEP, which demonstrated the highest percentage of antinociceptive activity in both phases of the formalin test, was then subjected to the fractionation processes. 

### 2.3. Preparation of *M. calabura* Fractions

The most effective partition, PEP, was fractionated by vacuum liquid chromatography (VLC) using silica gel 60 (1.07747, Merck, Germany). The fractions were eluted by stepwise elution using hexane and ethyl acetate and finally with methanol to give thirty-five (35) fractions. Analytical thin layer chromatography (TLC) on silica gel 60 F_254_ plates (Merck, Germany) was used to identify similar fractions. The fractions having the same chromatograms were combined and seven fractions were obtained. The fractions obtained, labelled as A, B, C, D, E, F, and G, were dissolved in 10% DMSO to the requisite dose (300 mg/kg) before being used and then subjected to the antinociceptive study.

### 2.4. Preparation of Drugs

Approximately 100 mg/kg of acetylsalicylic acid (ASA) (Bayer, Singapore) and 5 mg/kg morphine (Sigma, Germany) were used as positive controls. They were prepared in dH_2_O. All chemicals used in this study are of high quality standard. 

### 2.5. Experimental Animal

The present study was conducted using experimental Sprague-Dawley male rats weighing 200–250 g (age between 5 and 7 weeks old). The animals were obtained from the Animal Unit, Faculty of Pharmacy, Universiti Teknologi MARA, Malaysia, and cared according to the procedures described by Zimmermann [13] and adopted by Sani et al. [12]. All experiments were conducted between 0930 and 1830 to minimize the effects of environmental changes.

### 2.6. Toxicity Test

Acute toxicity test was conducted using oral fixed dose procedures based on Organisation for Economic Cooperation and Development (OECD) guidelines No. 420 [14]. The animals were fasted overnight and samples (5000 mg/kg) were administered orally at 10 mL/kg. Four hours after administration of test solutions, the animals were observed for mortality, if any, for 7 days.

#### 2.6.1. Biochemical Studies

Blood samples collected from all animals were each placed in a plain tube and allowed to clot for 45 min at room temperature. Serum were collected by centrifugation at 6,000 ×g for 10 min and stored at −80°C until analysis. Serum alanine aminotransferase (ALT), aspartate aminotransferase (AST), and alkaline phosphatase (ALP) were analyzed using ILab 300 Plus (Straits Scientific, USA).

### 2.7. Antinociceptive Test

#### 2.7.1. Formalin Test

The formalin test described by Rosland et al. [15] was used with little modification as described by Sani et al. [12]. Briefly, each rat was orally administered with the respective test solutions (vehicles (dH2O or 10% DMSO), positive controls (100 mg/kg ASA or 5 mg/kg morphine), or *M. calabura *(extract (MEMC), partitions (PEP, EAP or AQP), fractions, and isolated compounds) followed 30 min later by the administration of 50 *μ*L of 5% formalin to induce pain in the subplantar region (intraplantar; i.pl.) of the right hind paw. The rats were then individually placed in an inspection chamber which is a translucent plexiglas cage. Immediately after the phlogistic agent administration, the amount of time that the animal spent licking the injected paw, considered as an indicator of pain, was recorded in two phases, namely, the early (0–5 min) and late (15–30 min) phases, for the duration of 30 min.

### 2.8. Isolation of Active Compounds

After we had successfully determined the antinociceptive potential of all fractions, further procedures to collect the most effective fraction, namely, Fraction D, were carried out. The most effective fractions, indicated by their ability to produced the highest antinociceptive activity in both phases of the formalin test, were then subjected to the procedures of isolation and identification of bioactive compounds. Isolation and purification of bioactive compounds were performed using the radial chromatography eluted with hexane and increasing percentage of acetone (1%, 2%, and 5%). Each compounds collected were then chromatographed onto the TLC plate (Silica gel 60 F_254_, (Merck, Germany)). Four pure compounds were isolated from Fraction D and the spectral data of these pure compounds were obtained as follows: Infrared (IR) spectra were performed on FT-IR spectrometer (Varian 640-IR, Varian Australia PTY LTD, Australia). The mass spectra (MS) were measured using LCMS-TOF (6224 TOF LC/MS, Agilent Technology, USA). ^1^H NMR and ^13^C NMR spectra were recorded on Avance Bruker 300 MHz Spectrometer with measurement at 300 and 75 MHz for ^1^H and ^13^C NMR, respectively. Chemical shifts are reported in ppm and *δ* scale and the coupling constant are given in Hz. Following their identification, those pure compounds were dissolved in 10% DMSO to the requisite doses (50 and 100 mg/kg) and then submitted to the antinociceptive study.

### 2.9. Statistical Analysis

The results were expressed as mean ± S.E.M. One way analysis of variance (ANOVA) were performed and sequential differences for the means were calculated at the level of *P* < 0.05 using the post-hoc Dunnett test.

## 3. Result

### 3.1. Percentage Yield of *M. calabura *Extract, Partitions, and Fractions

Table 1 shows the percentage of yield of MCME, the partitions and fractions after their respective extraction procedure.

### 3.2. Toxicity Test and Biochemical Analysis

Study on toxicity of the MCME at the highest dose of 5000 mg/kg (OECD guideline No. 420) showed no mortality or any deleterious effects until day 14 after the extract administration. Therefore, the LD_50_ value by oral route could not be determined as no lethality was observed in rats up to 5000 mg/kg. Assessment of the liver function tests for rats pretreated with normal saline or 10% DMSO, or MCME revealed that the liver enzymes assessed, namely, ALT, AST, and ALP, did not show any significant differences (*P* > 0.05) when compared between groups (data not shown). These findings were supported by the histological findings, wherein pretreatment with MEMC did not cause any change to the structure of the liver (data not shown). Based on the acute toxicity study, three different doses (100, 500, and 1000 mg/kg) were prepared and used for further pharmacological studies.

### 3.3. Antinociceptive Activity of *M. calabura* Extract and Partitions

Table 2 shows the antinociceptive property of *M. calabura*'s extract (MEMC) and partitions (PEP, EAP, and AQP) at the doses regime of 100, 500, and 1000 mg/kg, respectively. PEP and EAP exhibit dose-independent antinociceptive activity in both phases of nociception, while AQP did not show antinociceptive activity in the early phase at all doses used with slight antinociception observed in the late phase. At the dose of 1000 mg/kg, PEP and EAP showed no significant differences in their intensity of antinociception when compared to morphine (5 mg/kg) in the first phase, while in the second phase, PEP showed better antinociceptive activity (*P* ≤ 0.05) when compared to aspirin (100 mg/kg). Higher doses of PEP, EAP, and AQP (1000 mg/kg) gave 74.5, 68.4, and 6.9% of nociceptive inhibition in the first phase of formalin test, respectively; while in the second phase, PEP, EAP, and AQP showed 89.1, 76.0%, and 26.9% % of nociceptive inhibition (Table 2).

### 3.4. Antinociceptive Activity of PEP Partition

PEP exhibited the highest antinociceptive activity in both phases of the formalin test when compared to EAP and AQP. Therefore, PEP was further partitioned using the VLC technique to yield seven fractions labeled as A, B, C, D, E, F, and G. All the fractions were subjected to the formalin test at the dose of 300 mg/kg. Of all fractions, only fraction D showed significant (*P* ≤ 0.05) antinociceptive activity in both phases of the formalin test. Fraction D (300 mg/kg) and morphine (5 mg/kg) do not show significant difference in the first phase of formalin test. However, in the second phase, fraction D showed lower antinociceptive property when compared to morphine (5 mg/kg), but did not show significant difference when compared to aspirin (100 mg/kg) (Table 3).

### 3.5. Isolation of Bioactive Compounds and Elucidation of Their Structure

Isolation and purification of fraction D, which was the most effective fraction in both phases of the formalin test, yielded four pure compounds. 

#### 3.5.1. Chemical Elucidation of Compounds **1–4**

Compound **1**: Yellow solid. IR: *ν*_max⁡_ (cm^−1^) 3434, 2987, 1638 ^1^H NMR (CDCL_3_, 300 MHz) *δ*_H_ 8.17 (2H, m), 7.56 (3H, m), 6.45 (1H, s), 3.99 (3H, s, OCH_3_), 3.93 (3H, s, OCH_3_), 3.89 (3H, s, OCH_3_), 12.623 (1H, OH). ^13^C-NMR (CDCL_3_, 75 MHz) *δ*_C_ 179.2 (C-4), 158.5 (C-2), 139.4 (C-3), 148.6 (C-5), 155.8 (C-7), 128.9 (C-8), 157.4 (C-9), 95.5 (C-6), 128.4 (C-2′ and C-6′), 128.7 (C-3′ and C-5′), 131.1 (C-4′), 105.4 (C-10), 130.6 (C-1′), 61.6 (OCH_3_), 60.3 (OCH_3_), 56.9 (OCH_3_). LCMSTOF *m/z* 327.0869 [M-1]^−^. 

Compound **2**: Yellow needle crystals. IR: *ν*_max⁡_ (cm^−1^) 3364, 1638, 1504, 1461, 1196, 782, 680, ^1^H NMR (CDCL_3_, 300 MHz) *δ*_H_ 6.37 (1H, d, *J* = 2.1 Hz), 6.46 (1H, d, *J* = 2.1 Hz), 8.08 (2H, m), 7.53 (3H, m), 3.88 (6H, s, OCH_3_), 12.65 (1H, b, OH). ^13^C-NMR (CDCL_3_, 75 MHz) *δ*_C_ 178.9 (C-4), 155.9 (C-2), 139.6 (C-3), 162.0 (C-5), 165.5 (C-7), 156.8 (C-9), 97.9 (C-6), 92.2 (C-8), 128.3 (C-2′ and C-6′), 128.6 (C-3′ and C-5′), 130.4 (C-4′), 106.1 (C-10), 130.9 (C-1′), 60.4 (3-OCH_3_), 55.8 (7-OCH_3_). LCMSTOF *m/z* 297.0768 [M-1]^−^.

Compound **3**: Yellow needle crystals. IR: *ν*_max⁡_ (cm^−1^) 3433, 2988, 1634, 1265 ^1^H NMR (CDCL_3_, 300 MHz) at *δ*_H_ 13.58 (1H, s, OH), 6.59 (1H, d, *J* = 8.7 Hz), 7.67 (1H, d, *J* = 9.0 Hz), 4.04 (3H, s, OCH_3_), 7.67 (2H, m), 7.45 (3H, m). ^13^C-NMR (CDCL_3_, 75 MHz) *δ*_C_  192.5 (C=O), 134.3 (C-3′), 155.3 (C-4′), 157.7 (C-2′), 134.7 (C-1), 115.0 (C-1′), 126.3 (C-6′), 120.1 (C-*α*), 128.6 (C-2 and C-6), 129.0 (C-3 and C-5), 130.7 (C-4), 106.5 (C-5′), 144.6 (C-*β*), 60.8 (3-OCH_3_). LCMSTOF *m/z *270.0911 [M-1]^−^.

Compound **4**: Orange crystals. IR: *ν*_max⁡_ (cm^−1^) 3345, 2926, 1663, 1957. ^1^H NMR (CDCL_3_, 300 MHz) *δ*_H_ 6.69 (1H, s), 6.53 (1H, d, *J* = 2.4), 6.40 (1H, d, *J* = 2.4), 7.91 (2H, m), 7.55 (3H, m), 3.91 (3H, s, OCH_3_) ^13^C-NMR (CDCL_3_, 75 MHz) *δ*_C_ 164.0 (C-2), 105.9 (C-3), 182.6 (C-4), 92.7 (C-5), 165.6 (C-6), 98.2 (C-7), 162.2 (C-8), 157.8 (C-9), 105.7 (C-10), 131.3 (C-1′), 126.3 (C-2′ and C-6′), 129.1 (C-3′ and C-5′), 131.8 (C-4′), 56.8 (6-OCH_3_). LCMSTOF *m/z* 267.0335 [M-1]^−^.

#### 3.5.2. Identification of the Bioactive Compounds

**Figure 2:**
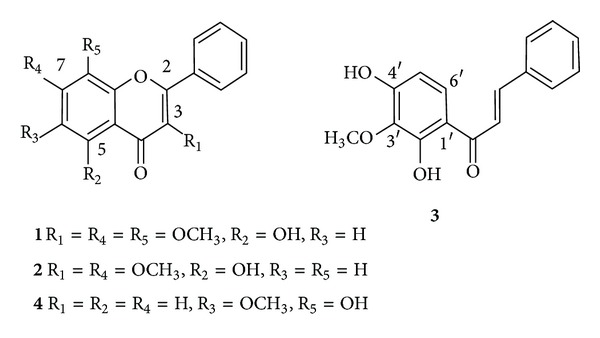
Chemical structures of compounds **1–4**.

A new flavone, calaburone (**4**) as well as three known compounds, 5-hydroxy-3,7,8-trimethoxyflavone (**1**) [16], 3,7-dimethoxy-5-hydroxyflavone (**2**) [17], and 2′,3′-dihydroxy-4′-methoxychalcone (**3**) [18] were identified from fraction D. Chemical structure of compounds **1–4** was shown in Figure 2.

Compound **4** (33 mg) was obtained as an orange crystal. The IR spectrum exhibited absorption band at 3345, 1663, and 1957 cm^−1^, indicating the presence of hydroxyl group (OH), unsaturated carbonyl group (C=O), and phenol group (C-O), respectively. A molecular formula of C_16_H_12_O_4_ was assigned for **4 **based on protonated molecular ion peak at *m/z* 267.0335 [M-H]^−^ in its HRESI-MS. The ^1^H NMR spectrum of **4 **showed the signals of monosubstituted benzene ring resonated at *δ*_H_ 7.91 (2H, m, H-2′, and H-6′) and *δ*_H_ 7.55 (3H, m, H-3′, H-4′, and H-5′), one methoxyl group at *δ*_H_ 3.91 (3H, s, 6-OCH_3_), two metacoupled protons at *δ*_H_ 6.53 (1H, d, *J* = 2.4 Hz, H-5) and 6.40 (1H, d, *J* = 2.4 Hz, H-7) and one signal of olefinic proton at *δ*_H_ 6.69 (1H, s, H-3).

14 signals were observed on the ^13^C APT NMR spectrum of **4** representing 16 carbons, confirming the basic skeleton of a flavone. Five signals at the downfield region consist of one carbonyl group resonated at *δ*_C_ 182.6 (C-4), and four oxyaryl carbons at *δ*_C_ 165.6 (C-6), 162.2 (C-8), 157.8 (C-9) and 164.0 (C-2). The signals of two quaternary carbons of C-1′ and C-10 were observed at *δ*_C_ 131.3 (C-1′) and 105.7 (C-10), respectively. The common carbons signals that appeared at *δ*_C_ 56.8 (6-OCH_3_) belong to the methoxyl group. The rest of the signals resonated at *δ*_C_ 105.9 (C-3), 92.7 (C-5), 98.2 (C-7), 126.3 (C-2′ and C-6′), and 129.1 (C-3′ and C-5′) and 131.8 (C-4′) represented eight methine carbons. This observation suggested that compound **4** is a flavone with unsubstituted B ring. Since only one methoxyl group was evident in both ^1^H and ^13^NMR spectra, the other group should be a hydroxyl group.

**Figure 3:**
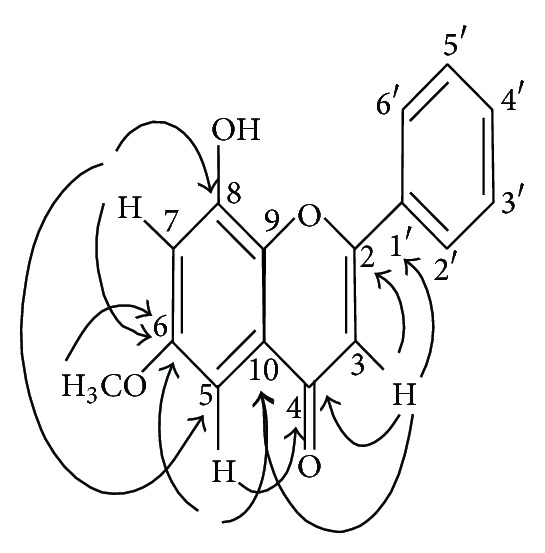
HMBC Correlation of 8-hydroxy-6-methoxyflavone (**4**).

The B-ring showed clearly its monosubstitution feature, thus suggesting that the methoxyl group, a pair of metacoupled, and an olefinic protons should be attached to A- and C-rings. The exact position of methoxyl group in **4** was determined from HMBC correlation. The ^3^ *J* correlations between the methoxyl (*δ*_H_ 3.91) and one of the metacoupled proton (*δ*_H_ 6.53) with *δ* 165.6 (C-6) indicates the attachment of the methoxyl group to C-6. This also leads to the assignment of a hydroxyl group to C-8 (Figure 3). NOESY spectrum of **4** showed correlations between methoxyl group (*δ*_H_ 3.91) with *δ*_H_ 6.53 (H-5) and *δ*_H_ 6.40 (H-7) which further confirmed the position of methoxyl group at C-6. The structure of **4** was thus assigned as shown in (Figure 2). Comparison with the previous study showed no similarity to this compound. Based on the assignments, this new flavone was established as calaburone, an 8-hydroxy-6-methoxyflavone.

### 3.6. Antinociceptive Activity of the Isolated Compounds

The antinociceptive property of isolated compounds was also evaluated using the formalin test at the dosage of 50 mg/kg (Table 4). Compound **3 **showed the higher percentage of nociceptive inhibition both at the first phase (34.5%) and second phase (43.8%) of the formalin test when compared to other pure compounds at the dose of 50 mg/kg.

Based on the antinociceptive test, the most significant activity was shown by **3**, followed by **4**, **2**, and **1**, respectively, both in first phase and second phase of the formalin test at the dose of 50 mg/kg. There is no structure-activity correlation sufficient to serve as working hypothesis to explain the antinociceptive activity between flavonoid derivative compounds (chalcone and flavones) isolated from *M. calabura* leaves. However, from the results obtained, the most potent antinociceptive activity is observed in compound that is more hydroxylated and less methoxylated than their less active analogue.

## 4. Discussion

Several papers have reported on the antinociceptive potential of *M. calabura *and several attempts have been made to determine the possible mechanism of antinociception involved as cited earlier. However, no attempt or report has been made to identify the bioactive compounds responsible for the antinociceptive activity of this plant. We have earlier reported on the MEMC antinociceptive activity [12] and determined several possible mechanisms of antinociception that might play a role in modulating the observed activity. 

In the present study, the formalin test was selected as the antinociceptive assay, as this model of nociception could be used to investigate on the ability of new compounds/extracts to exert peripheral and/or central antinociceptive effects. This is due to the assay biphasic characteristics, labeled as the early and late phases, which occur as a result of formalin administration [19]. The early phase, which persists for 5 min (0–5 min) immediately after the administration of formalin, is a neurogenic pain resulting from an acute response towards direct action of formalin on nociceptors within the intraplantar region. Meanwhile, the late phase, which appears between 15 and 60 min after the phlogistic agent administration, is considered as an inflammatory-mediated pain resulting from a tonic response due to the release of inflammatory mediators [20] and activation of the neurons in the dorsal horns of the spinal cord [21, 22]. Other advantages of this assay include its ability to verify the potential of new compounds/extracts to affect noninflammatory or inflammatory-associated pain. Generally, the peripherally-acting drugs inhibit only the late phase while the centrally-acting drugs inhibit both phases of the formalin test. The reason for choosing this assay could also be related to the report made by Rajendran et al. [23] that flavones, which are one of the subgroup within flavonoid-based compounds, inhibit both phases of the test. Interestingly, flavonoids, in general, and flavones, in particular, have been the main constituents of *M. calabura*.

Based on our findings, the MEMC inhibited the early and late phases of formalin test as reported by Sani et al. [12]. This finding leads us to partition the MEMC into PEP, EAP, and AQP in our attempt to isolate the responsible bioactive compounds according to their polarity. Upon subjection to the formalin test, PEP exerted the most effective antinociceptive activity and, therefore, was subjected to the fractionation processes, Of the seven fractions obtained from PEP, three fractions, namely, fraction C, D, and E, demonstrated antinociceptive activity in both phases of the formalin test. Fraction D was chosen for further isolation and identification processes as it shows the most effective antinociceptive activity in both phases of the formalin assay. 

Following the isolation and identification processes, four compounds, namely, three flavones (e.g., 5-hydroxy-3,7,8-trimethoxyflavone (**1**), 3,7-dimethoxy-5-hydroxyflavone (**2**), and 8-hydroxy-6-methoxyflavone (**4**)) and one chalcones (e.g., 2′,3′-dihydroxy-4′-methoxychalcone (**3**)) were identified, wherein compound **4** has been confirmed as a new compound and labeled as calaburone A, upon their subjection to formalin test, at the doses of 50 and 100 mg/kg, the antinociceptive activity of those compounds was observed in the increasing intensity as follows: 1 < 2 < 4 < 3. Only compound **3 **and **4** exerted good antinociceptive activity in both phases of the formalin test at both doses tested.

Flavonoids have been reported to be the main constituents of various parts of *M. calabura* [5, 6, 24, 25]. The leaves, in particular, have been reported to contain various types of flavonoids, namely, flavanones and flavones, as well as chalcones [6, 25]. In the present study, we successfully isolated three flavones and one chalcones as described above. Flavones have been shown to exert antinociceptive activity using the formalin test [23], and the ability of those identified flavones to inhibit formalin-induced nociception is concurrent with this report. Since the formalin test can discriminate pain into its central and peripheral components [26], the present study reveals the potential of MEMC, PEP, and EAP, and compounds **3** and **4** to act at the central level based on their ability to attenuate both phases of nociception [27]. Based on these findings, it is plausible to suggest that compounds **3** and **4** might, partly, contribute to the MEMC-induced antinociceptive as previously and currently observed. Interestingly, MEMC-induced antinociceptive activity has been shown to involve activation of the opioid receptors [12], whereas flavones, which are major constituents of *M. calabura *leaves [6, 25], have also been reported to induce antinociceptive activity via activation of the opioid receptors. Since flavones have been identified from MEMC, it is believed that they took part in the activation of opioid receptors. We were not able to determine the opioid receptors involvement in the antinociceptive activity of those pure compounds because the amount yielded was not enough for further studies. In term of chalcones, several reports have demonstrated that this class of compounds exhibited antinociceptive activity [28–30] even when assessed using the formalin test [30, 31]. Further preparations of extract and partitions for the isolation of those compounds are currently being carried out in our laboratory with hope of using them to study the possible mechanisms of antinociception involved. Based on our literature search, currently no approach has been made to determine the possible mechanisms of antinociception for both the flavones and chalcones. However, a report by Dawson and Snyder [32] that flavones derivatives modulate the proinflammatory gene expression, such as inducible NO synthase and cyclooxygenase-2, could be used to support our findings on the ability of the compounds to inhibit the late phase nociception. As described earlier, the late phase of the formalin test is mediated by the inflammatory processes.

NMR data for all the compounds isolated from fraction D shows that B-ring unsubstituted flavonoids are the major components of the leaves of *M. calabura* in this study. These results are in accordance with the leaves extract of *M. calabura* obtained from Peru [6]. However, only one compound, 3,7-dimethoxy-5-hydroxyflavone (**2**) was similarly being identified in both the Malaysian and Peruvian samples of *M. calabura*. Another three compounds (5-hydroxy-3,6,7-trimethoxyflavone (**1**), 2′,4′-dihydroxy-3′-methoxychalcone (**3**), and 8-hydroxy-6-methoxyflavone (**4**)) were newly isolated from this plant. This may represent a geographical difference, and/or a difference in site-specific accumulation of different metabolites. As up to date, no studies have ever reported on the antinociceptive property of the chemicals compounds isolated from *M. calabura *leaves, and that this is the first investigation on the antinociceptive property of the isolated compounds.

It has been claimed that flavonoid aglycones are able to pass through the gut wall and this is largely depending on the chemical structure of the flavonoids [33]. Once absorbed, flavonoids can influence many biological functions including protein synthesis, cell proliferation, differentiation, and angiogenesis for the benefit of mankind [33]. Since all the isolated compounds in this study are flavonoid aglycones, it can pass the gut wall to exert its antinociceptive and anti-inflammatory activities. Therefore, they can be easily formulated for oral administration. 

According to Peluso [34], many flavonoids are inhibitors of several isoforms of phosphodiesterase (PDE), and positions of hydroxylation are important for differential inhibition of PDE. For example, hydroxylation at the C-4′ position is important for inhibition of PDE_3_; while hydroxylation at the C-5 position is important for inhibition of PDE_1_, PDE_2_, PDE_4_, and PDE_5_ [34]. The competitive nature of the kinetics suggests that, flavonoids could compete for the same cAMP binding site in the adipocyte PDE [35]. Flavonoids are able to mimic cAMP stacking interaction, mainly because of the resemblance between the charge distributions of the pyronone rings of the inhibitors and the pyrimidine ring of cAMP, and/or resemblance between substrate and the inhibitors in their propensity to accept electron in a *π* (*pai*) orbital [36]. The elevation of intracellular cAMP has been associated with the inhibition of the various types of inflammatory cells including lymphocytes, monocytes, macrophages, neutrophils, eosinophils, mast cells, and basophils [37]. PDE_3_, PDE_4_, and PDE_7_ appear to be important in regulating cAMP in different cell types [37]. cAMP plays a major role in the inflammatory process. And recent researches had shown the involvement of PDEs in the mechanism of analgesic and inflammation [38]. 

In the present study, flavonoids isolated from *M. calabura* leaves extract contain hydroxyl group at the C-5 position (**1** and **2**). **3** has hydroxyl group attached at C-4′ and C-2′ position and **4** has hydroxyl group attached at C-8 position. We believed that, the antinociceptive and anti-inflammatory effect of the isolated compounds were due to the ability of the compounds to inhibit the production of PDEs, since flavonoids isolated from *M. calabura* leaves have the characteristic to become PDE inhibitor as described by Peluso [34]. In addition, the most potent PDE inhibitors were aglycones that had C-2 and C-3 double bond and a keto group at C-4 (characteristic for flavones and chalcone) [35]. However, opening C-ring of flavonoids such as chalcone are found to be more potent PDE inhibitors than other flavonoids that have an intact C-ring [35]. This may explain why **3 **gave more potent antinociceptive and anti-inflammatory effect in the present study.

Based on the acute toxicity study using a single dose (5000 mg/kg) of MEMC, the dosage range of MEMC for the antinociceptive study (e.g. 100, 500, and 1000 mg/kg) was selected based on the 50-, 10-, and 5- folds reduction of the dose 5000 mg/kg. Similar approach had been conducted by Freitas et al. [39] who used 1000 mg/kg as the highest dosage when administering the extract or fractions orally prior to assessment using the formalin test. From the results obtained, the MEMC and PEP, at 1000 mg/kg, exerted antinociceptive activity with similar effectiveness. This finding suggests that the amount of bioactive compounds present in the PEP was enough to produce antinociceptive activity at strength similar to MEMC. However, as the doses were reduced to 500 and 100 mg/kg, PEP antinociceptive activity decreased, particularly at the early phase, in comparison to MEMC. As for the fractions, the dose 300 mg/kg was selected based on a previous report [39] and finding that 500 mg/kg PEP exhibited approximately 40% analgesia in the early phase. Interestingly, the semipurified fraction D, at 300 mg/kg, caused analgesia at above 50% in both phases of the formalin test. The effectiveness of Fraction D was further explored, whereby the doses of the pure compounds obtained from that fraction were chosen to represent 6- and 3-fold reduction of the Fraction D. The reduction in antinociceptive potential of the four pure compounds in comparison to the semipurified Fractions D suggested that the former worked in synergism to induce remarkable antinociception as seen with the latter. Lastly, the pure compounds were tested at 50 and 100 mg/kg following recommendations published by Schmeda-Hirschmann and Yesilada [40]. 

In conclusion, the results in the present study support the folkloric use of the leaves of *M. calabura* against pain. The antinociceptive properties of the leaves of *M. calabura* are thought to be caused by the synergistic effects of the polyphenol compounds, which in this case were flavonoid derivatives. In this study, we managed to identify several flavonoids isolated from the leaves of *M. calabura *that are involved in its antinociceptive activity. We also have proposed several antinociceptive mechanisms involving flavonoids. However, further study still needs to be done to confirm the mechanism involved. It is noteworthy that compound **3** shows the potential to become antinociceptive drug with less side effects.

## Figures and Tables

**Table 1:** The dry weight and percentage of yield (%) of *M.  calabura* extract, partitions, and fractions.

Sample	End product	Types	Weight of sample (g)	Weight of end product (g)	Percentage of yield (%)
Dried leaves	Crude extract	MEMC	100	18.33	18.3

MEMC	Partition	PEP	550	139.0	25.3
		EAP	550	184.0	33.5
		AQP	550	227.0	41.2

PEP	Fraction	A	130	12.2	9.4
		B	130	33.4	25.7
		C	130	7.9	6.1
		D	130	6.8	5.2
		E	130	10.8	8.2
		F	130	15.9	12.2
		G	130	33.3	25.6

Fraction D	Pure compound	1	6.8	0.025	0.37
		2	6.8	0.041	0.61
		3	6.8	0.030	0.44
		4	6.8	0.033	0.49

**Table 2:** The antinociceptive profile of orally administered *M.  calabura* extract and partitions assessed using the formalin test in rats.

Treatment	Dose (mg/kg)	Early phase	Percentage of analgesia (%)	Second phase	Percentage of analgesia (%)
dH_2_O	—	70.7 ± 4.6	—	138.2 ± 3.2	—

10% DMSO	—	83.2 ± 2.7	—	149.0 ± 2.7	—

Morphine	5	15.2 ± 1.8^a^	78.6	7.2 ± 0.9^u^	95.2

Aspirin	100	49.8 ± 2.7^b^	29.5	17.7 ± 2.7^v^	88.2

MEMC	100	38.3 ± 3.9^c^	50.3	67.5 ± 5.7^w^	46.9
	500	27.3 ± 2.3^d^	64.6	45.7 ± 1.6^x^	64.1
	1000	20.0 ± 1.8^e^	74.1	25.7 ± 2.6^v^	79.8

PEP	100	63.5 ± 1.6^f^	22.4	85 ± 1.5^y^	41.9
	500	47.7 ± 5.9^c^	41.8	39.0 ± 5.4^x^	73.3
	1000	20.8 ± 0.5^e^	74.5	16.0 ± 1.4^v^	89.1

EAP	100	69.0 ± 1.9^c^	15.7	100.2 ± 1.3^z^	31.5
	500	59.2 ± 6.1^c^	27.7	75.3 ± 7.6^y^	48.4
	1000	25.8 ± 1.4^d^	68.4	35.2 ± 1.3^x^	76.0

AQP	100	71.7 ± 1.3	2.9	129.5 ± 2.3	6.3
	500	70.3 ± 3.1	4.7	105.5 ± 3.8^z^	23.6
	1000	68.7 ± 1.2	6.9	101.0 ± 1.6^z^	26.9

^a,b^Data with different superscript differed significantly (*P* < 0.05) when compared to the dH_2_O-treated group in the first phase.

^c,d,e,f^Data with different superscript differed significantly (*P* < 0.05) when compared to the DMSO-treated group in the first phase.

^u,v,z^Data with different superscript differed significantly (*P* < 0.05) when compared to the dH_2_O-treated group in the second phase.

^w,x,y,z^Data with different superscript differed significantly (*P* < 0.05) when compared to the DMSO-treated group in the second phase.

Values are mean ± S.E.M of 6 animals.

**Table 3:** The antinociceptive profile of seven fractions isolated from PEP that are derived from MEMC administered orally and assessed using the formalin test in rats.

Treatment	Dose (mg/kg)	First phase (s)	Percentage of inhibition (%)	Second phase (s)	Percentage of inhibition (%)
10% DMSO		83.2 ± 2.7	—	149.0 ± 2.7	—
A	300	79.7 ± 1.6	2.6	145.7 ± 2.2	0.5
B	300	74.8 ± 1.9	8.6	137.8 ± 2.0*	5.8
C	300	57.2 ± 2.1*	30.1	86.2 ± 1.9***	41.1
D	300	27.7 ± 2.4***	66.2	27.2 ± 1.8***	81.4
E	300	42.2 ± 3.4**	48.5	77.8 ± 1.5***	46.8
F	300	81.8 ± 3.3	0.0	86.7 ± 1.8***	40.8
G	300	74.3 ± 1.4	9.2	143.8 ± 2.4	1.7

^∗,∗∗,∗∗∗^Data with different superscript differed significantly at *P* < 0.05, *P* < 0.01, or *P* < 0.001, respectively, when compared to the DMSO-treated group within the respective phase of the formalin test.

Values are mean ± S.E.M of 6 animals.

**Table 4:** The antinociceptive profile of pure compounds isolated from fraction D of PEP administered orally and assessed using the formalin test in rats.

Treatment	Dose (mg/kg)	First phase (s)	Percentage of inhibition (%)	Second phase (s)	Percentage of inhibition (%)
10% DMSO		83.2 ± 2.7	—	149.0 ± 2.7	—

1	50	81.2 ± 2.0	2.5	132.8 ± 3.2^w^	10.9
	100	68.7 ± 2.2^a^	17.4	129.3 ± 1.6^w^	13.2

2	50	73.2 ± 3.8	12.0	120.8 ± 3.6^x^	18.9
	100	64.7 ± 2.5^a^	22.2	89.5 ± 8.1^y^	39.9

3	50	54.5 ± 2.1^b^	34.5	83.7 ± 1.2^y^	43.8
	100	ND	—	ND	—

4	50	69.5 ± 2.2^a^	16.4	119.0 ± 2.0^x^	20.1
	100	47.0 ± 2.0^c^	43.5	63.8 ± 4.0^z^	57.2

^a,b,c^Data with different superscript differed significantly (*P* < 0.05) when compared to the DMSO-treated group in the first phase.

^w,x,y,z^Data with different superscript differed significantly (*P* < 0.05) when compared to the DMSO-treated group in the second phase.

Values are mean ± S.E.M of 6 animals.
